# Association Between Pre-Stroke Physical Activity Patterns Assessed by the Baecke Physical Activity Questionnaire and Functional Recovery After First Ischemic Stroke: A Barthel Index-Based Cohort Study

**DOI:** 10.3390/neurolint18100190

**Published:** 2026-10-10

**Authors:** Sladjana M Vasilić-Brasnjević, Miloš Z Maksimović, Hristina D Vlajinac, Andja Ćirković, Tijana Dimkić Tomić, Branko D Jakovljević, Sindi Z Mitrović, Suzana Dedijer Dujović, Aleksandra S Vidaković

**Affiliations:** 1 Clinic for Rehabilitation “Dr Miroslav Zotović”, Faculty of Medicine, University of Belgrade, 11000 Belgrade, Serbia; sledz031@gmail.com (S.M.V.-B.); tijanatomic1985@gmail.com (T.D.T.); sindizmitrovic@gmail.com (S.Z.M.); suzanadedijer@yahoo.com (S.D.D.); 2 Institute of Hygiene and Medical Ecology, Faculty of Medicine, University of Belgrade, 11000 Belgrade, Serbia; milos.maksimovic@med.bg.ac.rs (M.Z.M.); branko.jakovljevic@med.bg.ac.rs (B.D.J.); 3 Institute of Epidemiology, Faculty of Medicine, University of Belgrade, 11000 Belgrade, Serbia; kristiv@eunet.rs; 4 Institute of Medical Statistics and Informatics, Faculty of Medicine, University of Belgrade, 11000 Belgrade, Serbia

**Keywords:** ischemic stroke, physical activity, Barthel Index, Baecke questionnaire, functional recovery

## Abstract

**Background/Objectives**: This study investigated the association between pre-stroke physical activity (PA) assessed using the Baecke Physical Activity Questionnaire (BPAQ) and functional status after a first-ever ischemic stroke (IS). **Methods**: This prospective cohort study included 91 patients who had had their first-ever IS and were admitted for inpatient rehabilitation. Functional status was assessed using the Barthel Index (BI) at rehabilitation admission, discharge, and three months after discharge. Linear mixed-effects models (LMMs) were adjusted for age, sex, the National Institutes of Health Stroke Scale (NIHSS) score, and the number of comorbidities. Predictors of BI ≥ 91 were examined using multivariable logistic regression. **Results**: BI improved significantly over follow-up (*p* < 0.001). Pre-stroke PA was associated with BI in the adjusted LMM (*p* = 0.021), whereas the time-by-BPAQ interaction was not significant (*p* = 0.952). Initial BI was associated with subsequent BI scores (*p* < 0.001). The work and leisure-time indices were associated with BI (*p* = 0.033 and *p* = 0.030), whereas the Sport Index was not (*p* = 0.089). None of the domain-by-time interactions were significant. Higher BI at rehabilitation admission was associated with higher odds of BI ≥ 91 at discharge and three months after discharge (OR = 1.11, *p* < 0.001; OR = 1.05, *p* = 0.015). **Conclusions**: Pre-stroke PA assessed using the BPAQ was associated with functional status across the observed time points, but there was no evidence that it was associated with a different longitudinal trajectory of BI recovery.

## 1. Introduction

Ischaemic stroke (IS) is the most common type of stroke, with an incidence of 92.4 per 100,000 citizens globally [1]. Between survived individuals, 25 to 75% remain functionally dependent [1]. Functional dependence is one of the most serious epilogs of IS, which has a significant influence on the quality of life of individuals who survived and, for their families, is also a great challenge for the health care system [2,3].

Socio-economic status (education, employment, marital status), health behaviors (smoking habits, alcohol consumption, physical activity), and clinical characteristics of patients {hypertension (HTN), diabetes mellitus (DM), hyperlipoproteinemia (HLP)} are, according to some studies, important factors for developing IS [2,3,4,5,6,7], as well as for functional outcome after IS [8].

In patients with ischemic stroke, polyvascular atherothrombotic disease has been associated with higher rates of vascular recurrence and increased levels of inflammatory markers, suggesting a link between the extent of atherothrombotic disease and systemic inflammation [9].

Recent evidence suggests that if PA before the stroke occurs, it may influence the overall functional outcome, stroke severity, and survival after stroke [10,11], but existing findings are inconsistent. In addition, the vast majority of studies have assessed pre-stroke PA using only simplified instruments [10,11]. Few studies have made a difference between occupational, sport, and leisure-time PA domains [11,12,13,14]. The Baecke Physical Activity Questionnaire (BPAQ), which offers a thorough evaluation of habitual PA across occupational, sport, and leisure-time domains [15,16], enables a more detailed characterization of domain-specific pre-stroke PA patterns; however, it is scarce, and the prospective cohort studies examining the association between pre-stroke PA domains and long-term functional recovery assessed by the BI are still lacking [11]. Moreover, data focusing specifically on first-ever IS patients undergoing rehabilitation are limited [17].

The aim of the study was to investigate the association between pre-stroke PA patterns assessed by the BPAQ and functional recovery, at rehabilitation admission and three months after rehabilitation discharge, in patients after their first-ever IS.

## 2. Materials and Methods

**Figure 1:**
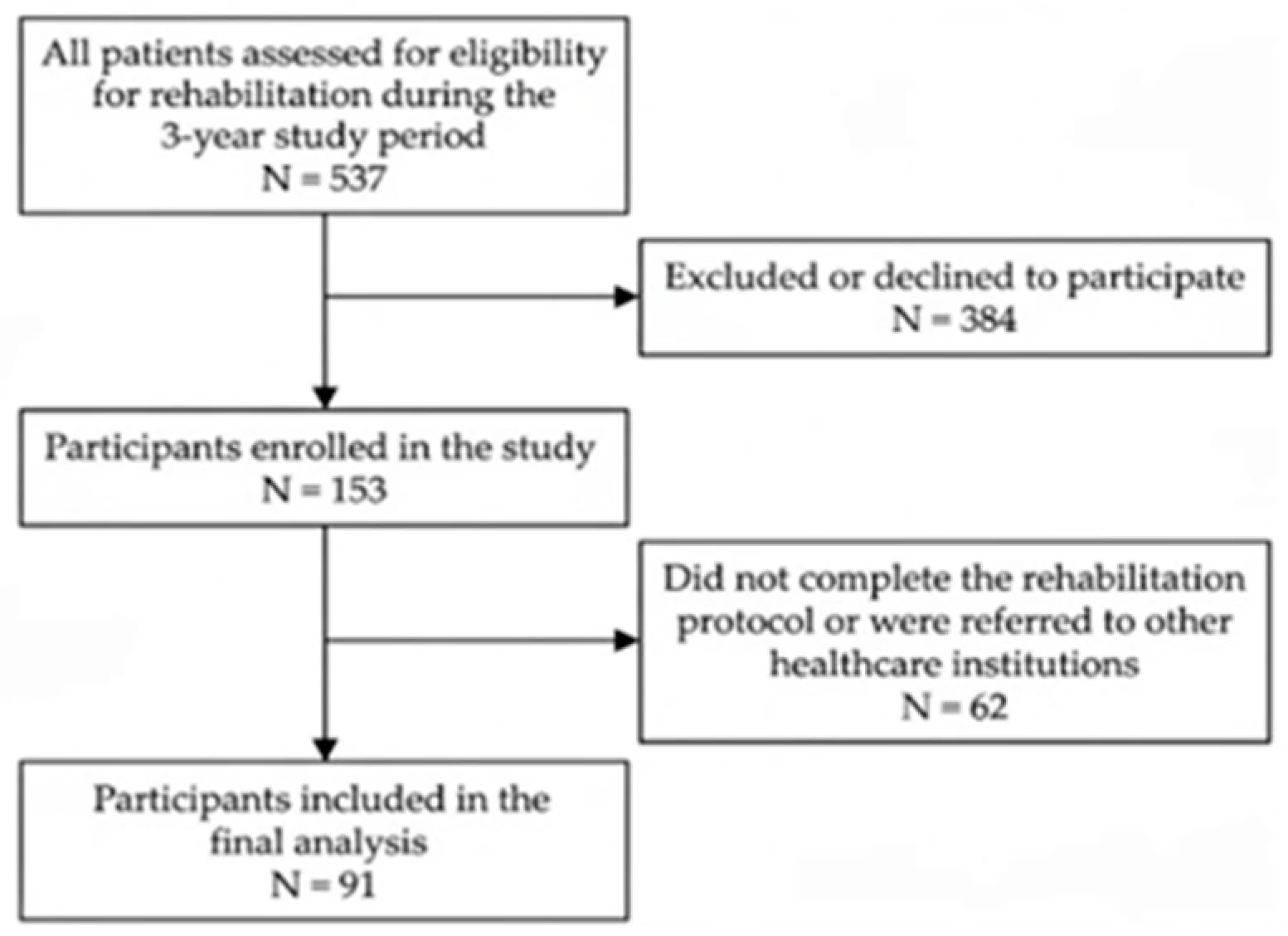
Flow diagram of patient selection.

This prospective observational study was conducted in Belgrade at the Clinic for Rehabilitation, “Dr Miroslav Zotović”, during 2022–2024. A total of 537 patients were assessed for eligibility. Of these, 384 did not meet the eligibility criteria or declined to participate. A total of 153 participants were enrolled in the study. Of these, 62 did not complete the rehabilitation protocol or were referred to other healthcare institutions, resulting in a final sample of 91 participants (Figure 1). It covered 91 patients of both sexes in the sub-acute phase of rehabilitation.

The diagnosis offirst-ever IS was based on clinical findings and neuroimaging methods (computerized tomography of the brain or brain magnetic resonance imaging (MRI). Included patients had first-ever IS and were over 18 years of age. All patients gave written consent to participate in the study. The exclusion criteria were brain tumor, head trauma, other central and peripheral neurological diseases, hip or knee diseases, and amputated limbs.

A structured questionnaire, specifically developed for this study, was used to collect data on age, sex, marital status, educational level, employment status, and health-related behavior (smoking status, alcohol consumption, and physical activity). Current smokers were defined as persons who smoke at least one cigarette a day or have stopped smoking within the previous year [18], and alcohol consumers were considered those patients who consume ≥ 1 alcoholic drink per month [19]. Also, data on comorbidities (HTN, DM, heart diseases, HLP) were collected. HTN was defined by a history of consumption of antihypertensive medication or by systolic blood pressure ≥ 140 mmHg, diastolic blood pressure ≥ 90 mmHg, or the presence of both at least two times in at least two separate measurements [20]. DM was defined by a history of diabetes confirmed by medical documentation of the patient or by usage of insulin or consumption of oral hypoglycemic medication [21]; heart disease was defined if there was any evidence of atrial fibrillation and myocardial infarction [22]. HLP was defined by a history of HLP confirmed by medical documentation or by statin usage for HLP treatment. Missing data were limited to three variables, educational level, marital status, and employment status, with two missing values for each variable.

Habitual physical activity was assessed using the Baecke Habitual Physical Activity Questionnaire (BPAQ), a standardized and validated instrument designed to evaluate usual PA. Patients were explicitly instructed to report their usual PA during the 12 months preceding their first-ever IS [16]. The questionnaire comprises three domains: work-related physical activity (Work Index), sports activity (Sport Index), and leisure-time physical activity excluding sports (Leisure-Time Index). The BPAQ was administered during the rehabilitation period, usually within the week preceding discharge. Patients completed the questionnaire independently whenever they were able to do so. When assistance was required because of motor impairment, aphasia/dysphasia, or difficulty understanding or completing the questionnaire, a physician read and explained the questions as necessary and recorded the patient’s responses. Family members did not complete the questionnaire or provide proxy responses; therefore, the reported PA was based on the patient’s own recollection and responses. The BPAQ was administered using a Serbian-language translation of the original questionnaire.

Data on clinical characteristics of the existing first-ever IS (type of stroke, National Institutes of Health Stroke Scale (NIHSS), hemiparesis/hemiplegia presence; aphasia/ dysarthria presence; and BI after IS) were collected from medical records.

The Barthel Index (BI), a standardized 10-item scale used to assess functional independence in activities of daily living, includes 10 categories: feeding, grooming, bathing, dressing, bladder control, bowel control, transfer from wheelchair to bed and vice versa, staircase mobility, toilet usage, mobility on flat surfaces (or wheelchair usage if walking is not possible). Overall score ranges from 0 (worst) to100 (best), where higher scores indicate greater independence. For interpretation of functional dependence, the BI scores were categorized as total dependence (0–20), severe dependence (21–60), moderate dependence (61–90), slight dependence (91–99), and independence (100), according to the commonly used classification proposed by Shah et al. [23]. For logistic regression analyses, BI ≥ 91 was used as the cutoff to represent a high level of functional independence, encompassing patients with slight dependence or complete independence.

Each question is separately scored depending on the level of independence of a patient when performing a certain activity or if the help of another person is needed. Patients with acute IS were initially treated in an acute neurological department where BI was assessed for the first time. They were subsequently transferred to the rehabilitation clinic. The rehabilitation process was carried out by a multidisciplinary team of experts that included a physiatrist, psychologist, speech therapist, physiotherapist, occupational therapist, and nurses. Acts of rehabilitation included kinesiotherapy, use of physical agents, occupational therapy, restitution of speech and language abilities, special care, and prevention of complications. The rehabilitation plan was individually adapted to each patient, and rehabilitation was carried out 5 days a week for 3 h a day [24].

Functional status was assessed using the BI at four time points: during the acute phase of first-ever IS treatment at the neurological department, at admission to the rehabilitation clinic, at discharge from the rehabilitation clinic, and three months after.

The timing of stroke onset was determined from the available medical documentation. Upon admission to the rehabilitation clinic, functional status was assessed for the second time. Patients subsequently underwent an inpatient rehabilitation program, after which the BI was reassessed at discharge. The follow-up time point was defined in relation to completion of the rehabilitation program. The assessment was performed three months after discharge from inpatient rehabilitation, as specified in the study protocol.

Complications during the inpatient rehabilitation stay were identified and recorded based on the patients’ medical records. The recorded complications included urinary tract infection, pneumonia, diarrhea, and shoulder pain.

Categorical data were presented as absolute and relative numbers in percentages. Numerical data were described with arithmetic mean and standard deviation or median and range from minimum to maximum, depending on the data distribution. Normality was assessed by mathematical (Shapiro-Wilk test) and graphical (histogram and box-plot) methods.

Firstly, in order to evaluate longitudinal changes in functional status, assessed by the BI, we performed a linear mixed-effects model (LMM). The BI was measured at each follow-up time point (at the beginning of the rehabilitation, i.e., at admission, at the end of rehabilitation, i.e., at discharge, and 3 months after the end of rehabilitation) and was specified as the dependent variable. Time was treated as a categorical fixed effect to account for the repeated measurements obtained at the three assessment time points. The fixed-effects component of the model included pre-stroke physical activity measured by BPAQ, the time-by-BPAQ interaction, and the baseline BI measured immediately after the first-ever ischemic stroke. The time-by-BPAQ interaction was included to assess whether the longitudinal change in the BI differed according to the level of pre-stroke PA. A subject-specific random intercept was included to account for the correlation between repeated observations within the same patient and to allow for individual differences in baseline BI values. Patient identification was therefore specified as the subject variable, whereas the time variable identified the repeated measurements within each patient. An unstructured covariance matrix was initially specified for the repeated measurements, allowing the variances and covariances between the three time points to be estimated without imposing a specific covariance pattern. Model fit was assessed using appropriate information criteria, including the Akaike information criterion (AIC) and Bayesian information criterion (BIC). The model parameters were estimated using restricted maximum likelihood (REML) for the final model. Fixed effects were evaluated using Type III tests of fixed effects. Estimated marginal means and pairwise comparisons between time points were used to characterize differences in the BIover time, with adjustment for multiple comparisons. Regression coefficients B, standard errors (SE), 95% confidence intervals of B, and *p*-values were reported. BI change during follow-up was assessed by Friedman’s test with the Wilcoxon signed-rank test for pairwise comparisons. To evaluate the change in frequencies in time periods, Cochrane Q for three time points and the McNemar test for pairwise comparisons were used.

In order to evaluate predictors of BI ≥ 91 (slight dependency) at discharge and 3 months after, we performed logistic regression analysis. Functional dependency was categorized according to the BI score as total dependency (0–20), severe dependency (21–60), moderate dependency (61–90), and slight dependency (91–99). Thus, all previously evaluated patient and disease characteristics were checked through univariable regression. Afterward, all statistically significant characteristics, at a level of 0.05, from univariable analysis, considering multicollinearity evaluated by the VIF method, were included in the multivariable model. Just the Barthel Index at discharge from the rehabilitation clinic had a VIF over 5; thus, it was excluded from the model, with BI ≥ 91 3 months after discharge as the outcome. The enter method was applied, and the odds ratio (OR), 95% confidence interval of the OR, and *p*-value were reported.

Three levels of physical activity were defined based on tertiles of the total score from the BPAQ and categorized as low, moderate, and high physical activity. Those groups were compared according to categorical variables using the chi-squared test or Fisher`s exact test, whereas for numerical variables, a one-way ANOVA and Tukey’s post hoc test were conducted, and the Kruskal-Wallis and Mann-Whitney U tests were applied for between-group comparisons.

All statistical methods were considered significant at the chosen level of 0.05. The analysis was performed in Statistical analyses were performed using IBM SPSS Statistics for Windows, version 29.0 (IBM Corp., Armonk, NY, USA) and R version 4.6.1 (R Foundation for Statistical Computing, Vienna, Austria) in RStudio version 2026.06.0 (Build 242).

## 3. Results

A total of 91 first-ever ischemic stroke patients, with an average age of 62.2 ± 12.1 years and a male-to-female ratio of 2:1, were enrolled in this prospective cohort study. Socio-demographic characteristics, comorbidities, and laboratory and hemodynamic parameters measured immediately after the first-ever IS of included patients are presented in Table 1. Two-thirds had primary or secondary education, were married, and were non-smokers, while less than half were employed and just 10% consumed alcohol. The most common comorbidity was HTN (87%), whilst the rarest was heart disease (19%).

The BI at the first-ever IS, at admission to the rehabilitation clinic, at discharge from the rehabilitation clinic, and three months after discharge were measured. Also, the BPAQ total score, Work Index, Sport Index, and Leisure-Time Index were registered in order to assess pre-stroke physical activity. Both scores are presented in Table 2. It was found that the BI increased over the follow-up period (*p* < 0.001), with the value at each subsequent time point being significantly higher than at the preceding time point (*p* < 0.001 for all pairwise comparisons).

**Figure 2:**
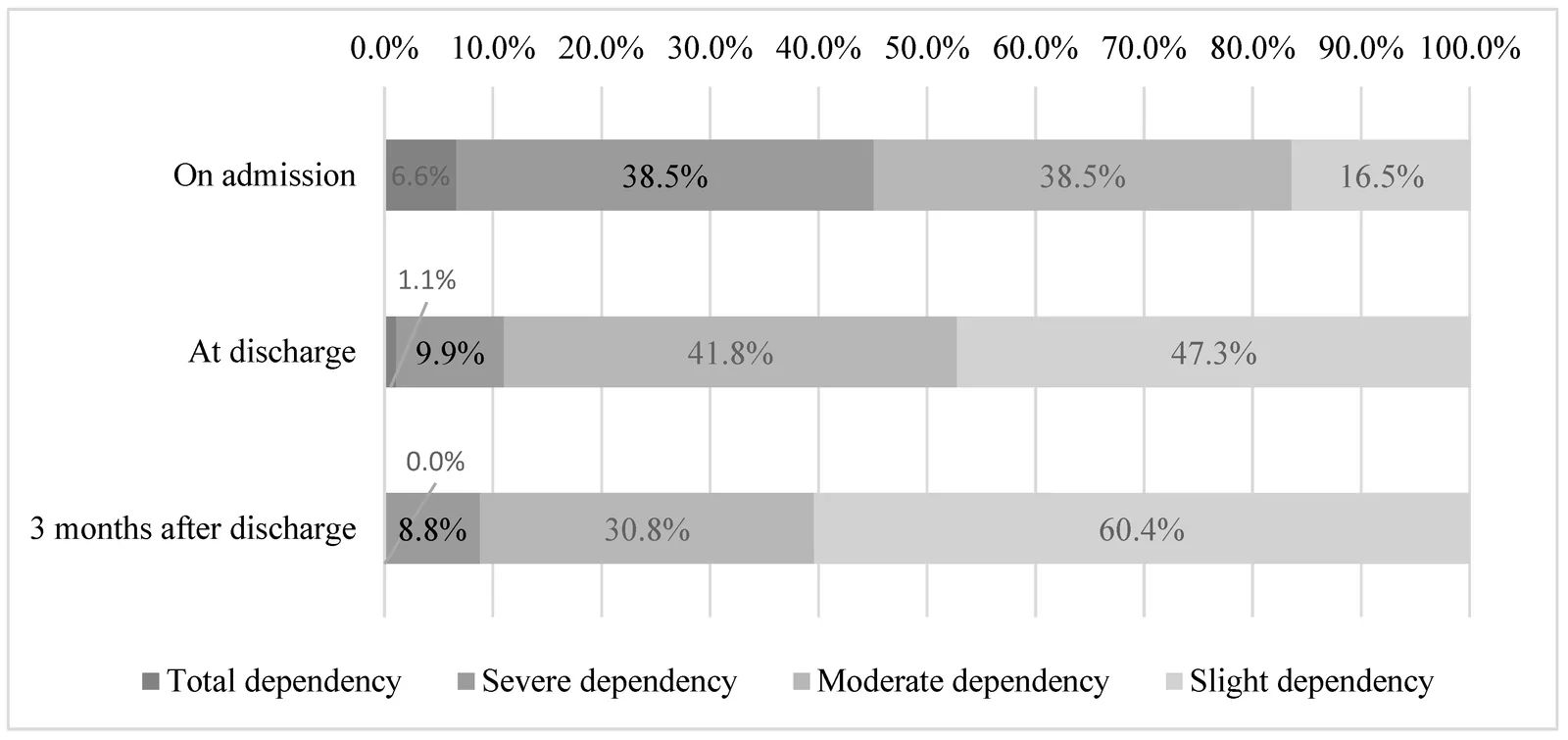
Disfunction level according to BI on admission, at discharge, and 3 months after discharge from the rehabilitation clinic.

Dysfunction level was defined as total, severe, moderate, and slight dependency according to BI value (0–20, 21–60, 61–90, ≥91, respectively). Distribution of dysfunction levels from admission to the rehabilitation clinic, through rehabilitation clinic discharge, up to 3 months after discharge is presented in Figure 2. There was a significant increase in the frequency of slight dependence (16%, 47%, 60%, *p* = 0.001) and a decrease in the frequency of total (7%, 1%, 0%, *p* = 0.006) and severe dependence (39%, 10%, 9%, *p* < 0.001) through the whole follow-up period. There was a significant increase in slight dependence between admission and discharge and between discharge and 3 months after discharge (*p* < 0.001 and *p* = 0.002). Also, there was a significant decrease in total dependence between admission and 3 months after discharge (*p* = 0.031), but there was no significant change between admission and discharge (*p* = 0.063) and between discharge and 3 months after discharge (*p* = 1.000). The frequency of severe dependency decreased between admission and discharge (*p* < 0.001), but not between discharge and 3 months after discharge (*p* = 1.000).

### 3.1. Association Between Pre-Stroke Physical Activity and Functional Recovery over Time

Firstly, in order to evaluate the impact of pre-stroke PA on longitudinal change in BI, used to express functional recovery, we performed an LMM with BI over time as the outcome (Table 3). The basic model included time, BPAQ, and initial BI evaluated at first-ever ischemic stroke as the fixed factors, and included patient ID a sa random factor. There was no statistically significant overall effect of time on the Barthel Index (*p* = 0.052). BPAQ was associated with the BI in the overall model (*p* = 0.024); however, at the reference time point (admission to the rehabilitation clinic), the estimated association between BPAQ and the BI was not significant (*p* = 0.092). There was no time-by-BPAQ interaction (*p* = 0.933), indicating that the association between pre-stroke physical activity and the BI did not significantly differ across the assessment time points. Specifically, the estimated differences in the BPAQ effect were non-significant both at discharge and three months after discharge, compared with admission (*p* = 0.918 and *p* = 0.827, respectively). Initial BI was independently and positively associated with subsequent BI scores (*p* < 0.001). After adjustment for sex, age, NIHSS score, and number of comorbidities, time became significantly associated with the BI (*p* = 0.034), with higher scores at discharge and three months after discharge compared with admission. BPAQ remained significantly associated with the BIin the overall model (*p* = 0.021). Also, the time-by-BPAQ interaction was still insignificant (*p* = 0.952), indicating no evidence that the longitudinal trajectory of functional status differed according to pre-stroke physical activity. Initial BI remained significantly associated with subsequent BI scores as in the unadjusted model (*p* < 0.001).

We evaluated the association between the three BPAQ domains separately (Work Index, Sport Index, and Leisure-Time Index) and longitudinal change in functional recovery after first-ever ischemic stroke (Table 4). In the two linear mixed models (Model A and Model B), longitudinal change in the Barthel Index was significantly associated with the Work Index (*p* = 0.009) and Sport Index (*p* = 0.011). Compared with admission, BI scores were higher at discharge and three months after discharge in all three models. Results of adjusted models showed that the Work Index and Leisure-Time Index were significantly associated with the BI (*p* = 0.033 and *p* = 0.030, respectively), whereas the association with Sport Index was not statistically significant (*p* = 0.089). None of the three domains showed a significant time-by-domain interaction, indicating no evidence that the longitudinal change in functional status differed according to pre-stroke physical activity in any BPAQ domain. The initial Barthel Index was consistently and positively associated with subsequent BI scores in all models (*p* < 0.001).

### 3.2. Association Between Pre-Stroke Physical Activity and Achieving Functional Independence (Barthel Index ≥ 91)

Although we found no clear significant association between pre-stroke PA and longitudinal changes in the BI, we sought to investigate factors associated with achieving slight dependence (BI ≥ 91 at discharge from the rehabilitation clinic), as well as with maintaining this level of functional status three months after discharge. Results of logistic regression analysis are presented in Table 5. Almost half of all the first-ever ischemic stroke patients achieved a BI score of 91 and over at discharge from the rehabilitation clinic, and even 60% of them achieved a BI score of 91 and over 3 months after discharge. Multivariable logistic regression models were adjusted for age and gender, with the total BPAQ score, Work Index, Sport Index, or Leisure-Time Index included as an additional covariate in the respective models. BI on admission to the rehabilitation clinic was consistently associated with higher odds of achieving functional independence at discharge (OR = 1.11, 95% CI 1.05–1.17, *p* < 0.001). A lower educational level was also associated with achievingthe outcome at discharge (OR = 4.22, 95% CI 1.05–16.93, *p* = 0.042). At three months after discharge, the BI on admission and Work Index remained independently associated with maintaining a BI ≥ 91 (OR = 1.05, 95% CI 1.01–1.10, *p* = 0.015 and OR = 0.17, 95% CI 0.03–0.91, *p* = 0.039, respectively).

### 3.3. Characteristics of Patients According to Pre-Stroke Physical Activity Level

Finally, to further characterize the study population according to the pre-stroke physical activity level, participants were categorized into tertiles of physical activity (low, moderate, and high), and socio-demographic and clinical characteristics were compared across these groups.

Table 6 presented socio-demographic characteristics of included patients according to three levels of physical activity: low (n = 31), moderate (n = 30), and high (n = 30). A significant difference in mean age was found depending on the level of physical activity (*p* = 0.032), with patients in the moderate activity group being older than those in the high physical activity group (*p* = 0.025). Also, patients with low in comparison with patients with moderate physical activity were commonly employed (*p* = 0.035).

The differences in the distribution of comorbidities and NIHSS score according to the level of physical activity are shown in Table 7. Hypertension was more frequent in patients with low and moderate physical activity compared to those with high physical activity (*p* = 0.005 and *p* = 0.020, respectively). The most common type of ischemic stroke was atherosclerotic (47%), with no difference in the distribution of any ischemic stroke types depending on the level of physical activity.

Laboratory parameters at the time of the first-ever ischemic stroke according to the level of physical activity are presented in Table 8. There was no difference in any of the evaluated parameters depending on the level of physical activity except in triglycerides, which were significantly higher in patients with low physical activity compared to those with high physical activity (*p* = 0.027).

Consequences, complications, and characteristics of physical rehabilitation are given in Table 9. Left and right motor deficit sides were equally distributed (55% vs. 45%); hemiparesis/hemiplegia were present in the majority of patients (97%), and aphasia/dysphasia were present in 29%. One fifth of patients experienced complications during hospitalization (22%). Early intrahospital rehabilitation started in almost all patients (97%), whilst rehabilitation in the rehabilitation clinic started more than 30 days later in one third of patients (28%). The median time of rehabilitation clinic stay was 40 days, with a minimum of 9 days and a maximum of 107 days. There was no difference in these characteristics depending on the level of physical activity of first-ever ischemic stroke patients.

Table S1 is showing disability level on admission, at discharge, and 3 months after discharge depending on the level of physical activity of first-ever ischemic stroke patients. The most patients on admission were moderately dependent (41%) with significant difference in distribution depending on the level of physical activity (*p* = 0.021). They were more common in patients with moderate than in low physical activity (*p* = 0.007). Patients who were slightly dependence on admission included 16% with significant difference depending on the level of physical activity (*p* = 0.024), and were more frequent in patients with low than with moderate physical activity (*p* = 0.012). On discharge, patients with slight dependence were common (47%), while there was a difference in distribution of patients with severe dependence according to the level of physical activity (*p* = 0.017), with greater frequency in patients with high than those with low and moderate physical activity (*p* = 0.012 and *p* = 0.038, respectively). Three months after discharge the largest portion of patients were slightly dependent (60%). There was no difference in the distribution of disfunction levels depending on the level of physical activity. Also, we evaluated the change of the number of disfunction levels in time from admission, to discharge, up to 3 months after discharge. The frequency of totally dependent patients did not change through the time. The number of severe dependent patients decreased between admission and discharge (*p* < 0.001) and between admission and 3 months after discharge (*p* < 0.001). Moderate dependency decreased from discharge to 3 months after it (*p* = 0.049). Number of slightly dependent patients increased between admission and discharge (*p* < 0.001), discharge and 3 months after it (*p* = 0.002), as well as from admission up to 3 months after discharge (*p* < 0.001).

## 4. Discussion

Applying the LMM showed that pre-stroke PA measured by BPAQ was significantly associated with BI in the adjusted model (*p* = 0.021), but no statistically significant association was observed between time and BPAQ scores (*p* = 0.952). This finding indicates that more physically active individuals enter the rehabilitation process with a more favorable capacity, but the speed of recovery itself occurs independently of pre-stroke PA. Similar results have been shown in some studies [25,26], and the explanation of this phenomenon lies in the concept of the brain reserve hypothesis [27], according to which regular PA before the formation of IS stimulates angiogenesis, synaptogenesis, and the expression of neurotrophic factors (BDNF)by mitigating the initial degree of damage to the brain and enabling the patient to start rehabilitation more favorably. Recent evidence suggests that higher levels of pre-stroke PA are associated with more favorable early and long-term functional outcomes after stroke, potentially through mechanisms related to neuroplasticity and vascular adaptation; however, direct causal evidence for accelerated neurophysiological recovery remains limited [28].

The strongest and most consistent independent predictor of the future functional status and of achieving functional independence (BI ≥ 91) was BI at admission (*p* < 0.001) in LMMs. The median BI increased significantly from 65.0 at admission to the rehabilitation facility as an inpatient, to over 90.0 at discharge and 95.0 three months after discharge (*p* < 0.001). The proportion of patients with mild dependence or complete functional independence (BI ≥ 91) increased from 16% at admission to 47% at discharge and 60% after three months (*p* = 0.001). These findings are consistent with previous studies showing that stroke subtype and stroke severity are significant determinants of post-stroke functional outcomes, with the greatest improvement in functional status [29]. Pathophysiologically, this result can be explained by the concept of neuronal reserve and initial penumbra damage [30]. The initial functional deficit directly reflects the volume of the infarct core and the degree of focal neurological outcome (median NIHSS in our cohort was 6). Buvarp et al. observed that favorable functional recovery was associated with increasing levels of physical activity across different degrees of stroke severity [31].

Multivariate analysis singled out the workplace activity index (Work Index, *p* = 0.033) and leisure-time activity index (Leisure-Time Index, *p* = 0.030) as statistically significant determinants of functional status, while the Sport Index had no statistical significance (*p* = 0.089). No significant statistical association with time was recorded in any domain. This observed negative association of the Work Index directly reflects the well-known “Physical Activity Paradox” in epidemiology [32]. Physical work at the workplace usually involves chronic monotonous workload, lifting loads, non-physiological body positions, insufficient rest periods, and increased systemic psychosocial stress. This type of activity leads to long-term HTN and sympathetic tone without activating the cardiovascular and metabolic training effect [32]. Conversely, leisure-time recreational PA boosts aerobic capacity, reduces systemic inflammation, and induces endothelial nitric oxide (NO) synthesis, thereby strengthening the neurovascular unit and cerebrovascular reserve [26]. Patients in the group with high PA had a significantly higher frequency of hospital complications (33.3% vs. 9.7% in the group with low activity, *p* = 0.024 and higher values of severe functional dependence at discharge (26.7% vs. 3.2% in the low group and 6.7% in the moderate group, *p* = 0.01 and *p* = 0.038)). In the group with high PA, the prevalence of cardioembolic first-ever IS was higher (30.0% vs. 12.9% in the low activity group). People with low PA were more frequently employed (53.3% vs. 26.7% in the moderate group; *p* = 0.035 and had more favorable basal BI already at admission (weak dependence in 29.0% of cases vs. 3.3% in the moderate group, *p* = 0.012)). This “healthy worker effect” created a more favorable starting position for the group with low PA. Patients in the group with moderate PA were significantly older (66.4 ± 10.1 years) compared to the high activity group (58.3 ± 14.6 years, *p* = 0.025). The older population (dominant in the moderate and low PA groups) had dramatically higher HTN (93.3% in the moderate and 96.8% in the low group vs. 70.0% in the high activity group, *p* = 0.005 and *p* = 0.020). From a pathophysiological point of view, the process of brain aging is characterized by a progressive decrease in the density of synapses, reduced secretion of neurotrophic factors (primarily BDNF-brain-derived neurotrophic factor), the presence of microvascular leukoaraiosis, and sarcopenia [33]. In elderly patients with long-term HTN (over 90% presence in the moderate/low group), chronic arteriolosclerosis reduces collateral cerebral blood flow, which increases the sensitivity of the penumbra to acute ischemia [30]. Despite the mentioned biological disadvantages, elderly patients from the group with moderate PA achieved extremely favorable functional recovery: at the three-month follow-up, as many as 63.3% of these patients reached the level of mild dependence/independence (BI ≥ 91), without a single patient in the category of complete dependence. This finding proves that continuous moderate PA in old age acts as a strong “buffer mechanism” (neurovascular reserve) that neutralizes the negative effects of advanced age and HTN. Even if moderate activity does not completely relieve acute neurological deficit in the first days of the stroke, it preserves microcirculation, preserves peripheral muscle mass, and improves cardiorespiratory fitness, allowing elderly patients to make maximum use of the potential of inpatient rehabilitation (the median duration of which in our study was 40 days).

Differences in age and work status between tertiles of total PA further demonstrate that the BPAQ score is not an isolated biological trait, but an indicator linked to a broader demographic and social context. Older people can have a lower Sports Index and Work Index due to retirement, chronic diseases, or previously reduced mobility, while working people can achieve a higher Work Index regardless of cardiorespiratory fitness. Therefore, based on the comparison of tertiles, it cannot be concluded that a certain level of PA directly causes better or worse recovery. In particular, it is not justified to claim that moderate PA in elderly patients “neutralized” the unfavorable influence of age or HTN, because brain reserve, collateral circulation, cardiorespiratory capacity, or neuroplasticity were not directly assessed in this study. Very elderly patients with first-ever IS, most commonly defined as persons aged ≥ 80 years, represent a clinically specific subgroup that differs from younger patients in terms of demographic characteristics, vascular risk factor profiles, burden of comorbidities, complications, and functional outcomes. In addition, the relative contribution of individual vascular risk factors may change with age. Future studies with adequate statistical power should therefore specifically examine whether the association of pre-stroke PA and functional recovery differs in very old patients, taking into account frailty, multimorbidity, severity, and post-stroke complications [34,35,36,37]. Zucchella et al. showed that very elderly stroke patients can achieve significant functional improvement during intensive rehabilitation despite their advanced age, although the magnitude of recovery may be lower than in younger older adults. They also found that age, stroke severity, and functional status at admission are important predictors of rehabilitation outcome [38].

Our findings should also be considered in the context of the characteristics of the study population. The mean age of the participants was 62.2 years, and approximately two-thirds of the participants were men. Patients differed with respect to pre-stroke PA, as well as in age and employment status observed between the low-, moderate-, and high-PA groups. Patients in the moderate-PA group were older than those in the high-PA group, whereas employment was more frequent in the low-PA group than in the moderate-PA group. These differences illustrate the multidimensional nature of habitual PA: occupational, sports, and leisure-time activities are influenced by age, employment, and other social and demographic characteristics. These findings further highlight the multidimensional nature of PA and the importance of considering its different domains separately.

This study also has several methodological strengths. First, it included only patients with a first-ever IS, thereby reducing the potential influence of previous stroke-related disability on the functional outcome. Second, pre-stroke PA was assessed using the BPAQ, allowing occupational, sports, and leisure-time activities to be considered separately rather than relying exclusively on a total PA score. Third, the longitudinal design enabled the repeated assessment of functional status during the acute phase after stroke, at admission to rehabilitation, at discharge from rehabilitation, and three months after discharge. Fourth, the use of linear mixed-effects models allowed repeated measurements within individual patients to be appropriately accounted for and allowed for an assessment of whether functional recovery over time differed according to the level of pre-stroke PA. Finally, the analyses were adjusted for clinically relevant factors, including age, sex, NIHSS, and comorbidity burden.

Several limitations should nevertheless be considered. First, the sample size was relatively small, with 91 participants, which limits statistical power, particularly for analyses of individual domains and multivariable logistic regression models. The relatively small sample size may also limit the ability to detect interactions between time and PA, which are particularly important for assessing differences in the longitudinal trajectory of functional recovery. Therefore, the possibility of type II error cannot be excluded, and statistically non-significant findings should be interpreted cautiously. Second, pre-stroke PA was assessed retrospectively after stroke. Therefore, recall bias is possible, particularly among patients with cognitive, language, or communication difficulties. To reduce this ambiguity, participants were specifically instructed that the BPAQ questions referred to their usual PA during the 12 months preceding the first-ever IS, rather than the 12 months preceding questionnaire administration. Third, the BI primarily assesses basic activities of daily living and may not capture higher levels of motor functioning, participation, or more subtle differences in functional recovery that could potentially be related to previous PA levels. A more comprehensive assessment of mobility, instrumental activities of daily living, or quality of life might identify associations that cannot be detected using the BI as the sole measure.

Another limitation is the relatively short follow-up period. The three-month assessment captures an important phase of early recovery but may not be sufficient to determine whether pre-stroke PA affects long-term adaptation, participation, maintenance of functional independence, or later functional decline. In addition, the observational design does not allow causal conclusions to be drawn. Associations between PA and clinical outcomes may be influenced by unmeasured factors, such as pre-stroke frailty, cognitive status, nutritional status, socioeconomic circumstances, or premorbid functional capacity. It should also be considered that the study was conducted in a single specialized rehabilitation facility and included patients admitted within six months after their first-ever IS. Consequently, the findings may not be generalizable to all patients with IS, particularly those with very mild stroke who do not require inpatient rehabilitation and those with severe stroke who are unable to participate in rehabilitation. Selection related to referral to a rehabilitation facility may therefore have influenced the characteristics of the study cohort.

The present findings have several implications for future research. Larger multicenter prospective studies should assess pre-stroke PA using validated multidimensional questionnaires and objective methods of measuring PA. Future studies should also incorporate premorbid functional status, frailty, cognitive status, stroke severity, lesion characteristics, and rehabilitation intensity. Longer follow-up would be useful to determine whether pre-stroke PA is associated with outcomes beyond basic activities of daily living, including mobility, instrumental activities of daily living, participation, quality of life, and maintenance of functional independence. Analyses of individual PA domains should also be retained, as occupational, sports, and leisure-time activity may have different relationships with post-stroke outcomes.

## 5. Conclusions

In conclusion, patients in this cohort demonstrated significant improvement in functional status from the acute post-stroke period through rehabilitation and three months after discharge. However, pre-stroke PA, assessed using the BPAQ, was not associated with a different longitudinal trajectory of recovery according to the BI. The absence of a different recovery trajectory was also observed for the occupational, sports, and leisure-time activity domains. Although some PA domains showed associations with BI or with specific functional outcomes in secondary analyses, these findings do not indicate that pre-stroke PA was associated with the magnitude of functional recovery. In contrast, initial functional status was consistently associated with subsequent BI values.

## Figures and Tables

**Table 1:** Socio-demographic characteristics, comorbidities, and laboratory and hemodynamic parameters of the first-ever ischemic stroke patients.

Socio-Demographic Characteristics		Comorbidities		Laboratory and Hemodynamic Parameters		Consequences	
Characteristic	Description	Comorbidity	Description	Parameter	Description	State	Description
Age (years) Mean ± sd	62.2 ± 12.1	HTN, n (%)	79 (86.8)	Glycemia, Mean ± sd	7.2 ± 2.2	Motor deficit side, n (%)	
Gender, n (%)		DM, n (%)	23 (25.3)	Total cholesterol, Mean ± sd	5.0 ± 1.6	Left	50 (54.9)
Males	56 (61.5)	Heart disease, n (%)	17 (18.7)	HDL, med (min–max)	2.1 (0.8–3.9)	Right	41 (45.1)
Females	35 (38.5)	HLP, n (%)	39 (42.9)	LDL, med (min–max)	2.3 (1.0–9.0)	Functional deficit (hemiparesis/hemiplegia), n (%)	88 (96.7)
Educational level, n (%)		Number of comorbidities, med (min–max)	2 (0–4)	Triglycerides, med (min–max)	1.4 (0.7–5.2)	Functional deficit (aphasia/dysphasia), n (%)	27 (29.7)
Primary and secondary	56 (62.9)	Ischemic stroke type, n (%)		STA, mean ± sd	138.3 ± 33.8	Complications during hospitalization, n (%)	20 (22.0)
Tertiary and more	33 (37.1)	Arteriosclerotic	41 (45.1)	DTA, mean ± sd	98.5 ± 20.9	Early rehabilitation in hospital, n (%)	88 (96.7)
Marital status, n (%)		Cardioembolic	19 (20.9)			Duration of early rehabilitation, med (min–max)	10 (0–48)
Married	61 (68.5)	Lacunar	10 (11.0)			Time from first-ever stroke to rehabilitation, med (min–max)	17 (1–1472)
Unmarried (divorced, widow, single)	28 (31.5)	Other	21 (23.1)			Rehabilitation clinic stay, med (min–max)	40 (9–107)
Employment status, n (%)		NIHSS, med (min–max)	6 (2–21)			Time from first-ever stroke to the end of rehabilitation, med (min–max)	71 (16–1503)
Employed	36 (40.4)					Rehabilitation clinic stay, med (min–max)	40 (9–107)
Unemployed	53 (59.6)						
Smoking habit, n (%)							
Smoker	29 (31.9)						
Non-smoker	62 (68.1)						
Alcohol abuse, n (%)							
Yes	13 (14.3)						
No	78 (85.7)						

Measured immediately after first-ever ischemic stroke. Abbreviations: HTN, hypertension; DM, diabetes mellitus; HLP, hyperlipidemia; NIHSS, National Institutes of Health Stroke Scale; HDL, high-density lipoproteins; LDL, low-density lipoproteins.

**Table 2:** Barthel Index and Baecke Physical Activity Questionnaire.

BI, Med (Min–Max)		*p* *	BPAQ, Med (Min–Max)	
At first-ever ischemic stroke	60.0 (0.0–95.0)	**<0.001**	Total score	5.75 (3.25–9.00)
At admission	65.0 (5.0–100.0)		Work score	1.63 (0.75–75)
At discharge	90.0 (10.0–100.0)		Sport score	1.75 (1.00–4.25)
3 months after discharge	95.0 (30.0–100.0)		Leisure-time score	2.00 (0.75–3.25)

* for the level of significance of 0.05 according to Friedman’s test, with Wilcoxon’s test used for pairwise comparisons, statistically significant *p*-values are shown in bold.

**Table 3:** Unadjusted and adjusted linear mixed models for longitudinal change in the Barthel Index as the outcome and pre-stroke physical activity assessed by the BPAQ as a factor.

Factor	Unadjusted				Adjusted
	B	SE	95%CI B	*p* *	*p* *
Time ^a^				0.052	**0.034**
Discharge vs. admission Barthel ^b^	18.55	7.80	3.06–34.05	0.019	**0.013**
3 months after discharge vs. discharge ^b^	21.54	8.74	4.17–38.91	0.016	**0.010**
BPAQ ^a^				**0.024**	**0.021**
BPAQ ^b^	−2.74	**1.61**	−5.95–0.46	0.092	0.108
Time * BPAQ ^a^				0.933	0.952
Time * BPAQ discharge vs. admission ^b^	0.13	1.27	−2.39–2.65	0.918	0.933
Time * BPAQ 3 months after discharge vs. discharge ^b^	0.31	1.42	−2.51–3.13	0.827	0.981
BI at first-ever stroke ^a^				**<0.001**	**<0.001**
BI at first-ever stroke ^b^	0.33	0.06	0.21–0.44	**<0.001**	**<0.001**

* for the level of significance according to linear mixed-effects model analysis. (a) Type III fixed-effect test. (b) Estimates of fixed-effect results. Statistically significant *p*-values are shown in bold.

**Table 4:** Unadjusted and adjusted linear mixed models for longitudinal change in the Barthel Index as the outcome and the pre-stroke physical activity assessed by the three BPAQ domains: Work Index, Sport Index, and Leisure-Time Index as factors.

Factor	Unadjusted				Adjusted
	B	SE	95%CI B	*p* *	*p* *
***MODEL A—Work index as factor***					
Time ^a^				**0.022**	**0.019**
Discharge vs. admission Barthel ^b^	16.01	6.02	4.05–27.97	**0.009**	**0.008**
3 months after discharge vs. discharge ^b^	19.06	6.74	5.66–32.45	**0.006**	**0.005**
Work index ^a^				**0.009**	**0.009**
Work index ^b^	−9.25	4.16	−17.53 to −0.97	**0.029**	**0.033**
Time * Work index ^a^				0.788	0.814
Time * Work index discharge vs. admission ^b^	1.89	3.28	−4.62–8.40	0.566	0.612
Time * Work index 3 months after discharge vs. discharge ^b^	2.47	3.67	−4.83–9.77	0.504	0.542
BI at first-ever stroke ^a^				**<0.001**	**<0.001**
BI at first-ever stroke ^b^	0.31	0.06	0.20–0.43	**<0.001**	**<0.001**
***MODEL B–Sport index as factor***					
Time ^a^				**<0.001**	**<0.001**
Discharge vs. admission Barthel ^b^	20.45	4.64	11.23–29.66	**<0.001**	**<0.001**
3 months after discharge vs. discharge ^b^	22.28	5.20	11.95–32.62	**<0.001**	**<0.001**
Sport index ^a^				**0.011**	**0.010**
Sport index ^b^	−5.24	2.89	−10.98–0.51	0.074	0.089
Time * Sport index ^a^				0.362	0.349
Time * Sport index discharge vs. admission ^b^	−0.58	0.28	−5.11–3.94	0.798	0.644
Time * Sport index 3 months after discharge vs. discharge ^b^	0.59	2.56	−4.49–5.67	0.817	0.973
BI at first-ever stroke ^a^				**<0.001**	**<0.001**
BI at first-ever stroke ^b^	0.33	0.06	0.21–0.44	**<0.001**	**<0.001**
***MODEL C—Leisure-time index as factor***					
Time ^a^				0.067	0.052
Discharge vs. admission Barthel ^b^	12.01	5.39	1.31–22.72	**0.028**	**0.022**
3 months after discharge vs. discharge ^b^	14.24	6.03	2.27–26.21	**0.020**	**0.015**
Leisure-time index ^a^				**0.049**	**0.047**
Leisure-time index ^b^	−7.38	3.25	−13.83 to −0.92	**0.026**	**0.030**
Time * Leisure-time indexa				0.278	0.333
Time * Leisure-time index discharge vs. admission ^b^	3.63	2.54	−1.42–8.69	0.157	0.196
Time * BPAQ 3 months after discharge vs. discharge ^b^	4.55	2.84	−1.10–10.20	0.114	0.144
BI at first-ever stroke ^a^				**<0.001**	**<0.001**
BI at first-ever stroke ^b^	0.33	0.06	0.21–0.44	**<0.001**	**<0.001**

* for the level of significance according to linear mixed-effects model analysis. (a) Type III fixed-effect test. (b) Estimates of fixed-effect results. Statistically significant *p*-values are shown in bold.

**Table 5:** Factors associated with the Barthel Index ≥ 91 at discharge and at 3 months after discharge from rehabilitation clinic.

Factor	Univariable Logistic Regression			Multivariable Logistic Regression			*p* ^a^	*p* ^b^	*p* ^c^	*p* ^d^
	OR	95%CI OR	*p* *	OR	95%CI OR	*p* *				
**At discharge**										
Educational level										
Tertiary or more	Ref.			Ref.			Ref.	Ref.	Ref.	Ref.
Primary and secondary	6.190	2.29–16.74	**<0.001**	4.219	1.05–16.93	**0.042**	**0.042**	**0.042**	**0.046**	**0.045**
NIHSS	0.796	0.68–0.94	**0.006**	1.040	0.84–1.29	0.724	0.728	0.750	0.726	0.680
Barthel Index immediately after first-ever stroke	1.049	1.02–1.06	**0.001**	0.990	0.95–1.03	0.602	0.661	0.609	0.662	0.729
Barthel Index at admission to rehabilitation clinic	1.109	1.07–1.15	**<0.001**	1.109	1.05–1.17	**<0.001**	**<0.001**	**<0.001**	**<0.001**	**<0.001**
**3 months after discharge**										
Educational level										
Tertiary or more	Ref.			Ref.						
Primary and secondary	2.533	1.05–6.14	**0.040**	1.935	0.45–8.34	0.376	0.501	0.376	0.318	0.494
NIHSS	0.720	0.60–0.86	**<0.001**	0.832	0.63–1.09	0.188	0.108	0.188	0.148	0.302
Complications during hospitalization	0.140	0.05–0.44	**0.001**	0.581	0.11–3.14	0.528	0.277	0.528	0.473	0.295
Duration of rehabilitation	0.981	0.96–0.99	**0.033**	1.009	0.98–1.04	0.558	0.311	0.558	0.391	0.649
Total cholesterol	1.500	1.01–2.23	**0.046**	1.544	0.94–2.55	0.089	0.182	0.089	0.146	0.126
DTA	1.036	1.01–1.06	**0.005**	1.025	0.99–1.07	0.196	0.190	0.196	0.136	0.163
Barthel Index immediately after first-ever stroke	1.033	1.01–1.06	**0.002**	0.992	0.96–1.03	0.672	0.635	0.672	0.592	0.683
Barthel Index at admission to rehabilitation clinic	1.067	1.04–1.10	**<0.001**	1.054	1.01–1.10	**0.015**	**0.020**	**0.015**	**0.020**	**0.019**
Barthel Index at discharge from rehabilitation clinic	1.316	1.17–1.48	**<0.001**							
Work Index	0.307	0.12–0.78	**0.013**	0.172	0.03–0.91	**0.039**	**0.010**	**0.039**	**0.037**	**0.010**

* for the level of significance of 0.05; (a) adjusted for age, gender, and BPAQ; (b) adjusted for age, gender, and Work Index (except for Barthel Index ≥91 3 months after discharge from rehabilitation clinic, this model was adjusted for age and gender); (c) adjusted for age, gender, and Sport Index; (d) adjusted for age, gender, and Leisure-Time Index, statistically significant *p*-values are shown in bold.

**Table 6:** Socio-demographic characteristics of first-ever ischemic stroke patients according to the level of physical activity.

Characteristic	Low PA n = 31	Moderate PA n = 30	High PA n = 30	*p* *	Low vs. Moderate	Low vs. High	Moderate vs. High
Age (years) Mean ± sd	61.9 ± 10.2	66.4 ± 10.1	58.3 ± 14.6	**0.032**	0.300	0.459	**0.025**
Gender, n (%)							
Males	17 (54.8)	22 (73.3)	16 (53.3)	0.209	0.133	0.906	0.108
Females	14 (45.2)	8 (26.7)	14 (46.7)				
Educational level, n (%)							
Primary and secondary	17 (56.7)	21 (70.0)	18 (62.1)	0.561	0.284	0.673	0.520
Tertiary and more	13 (43.3)	9 (30.0)	11 (37.9)				
Marital status, n (%)							
Married	20 (66.7)	23 (76.7)	18 (62.1)	0.465	0.390	0.712	0.223
Unmarried (divorced, widow, single)	10 (33.3)	7 (23.3)	11 (37.9)				
Employment status, n (%)							
Employed	16 (53.3)	8 (26.7)	12 (41.4)	0.108	**0.035**	0.358	0.233
Unemployed	14 (46.7)	22 (73.3)	17 (58.6)				
Smoking habit, n (%)							
Smoker	11 (35.5)	11 (36.7)	7 (23.3)	0.470	0.923	0.298	0.260
Non-smoker	20 (64.5)	19 (63.3)	23 (76.7)				
Alcohol abuse, n (%)							
Yes	4 (12.9)	5 (16.7)	4 (13.3)	0.933	0.731F	1.000F	1.000F
No	27 (87.1)	25 (83.3)	26 (86.7)				

* for the level of significance of 0.05 according to the one-way ANOVA and Tukey’s post hoc test; the chi-square test and Fisher’s exact test were used where appropriate. Abbreviations: PA, physical activity, statistically significant *p*-values are shown in bold.

**Table 7:** Comorbidities and NIHSS score of first ischemic stroke patients according to the level of physical activity.

Comorbidity	Low PA	Moderate PA	High PA	*p* *	Low vs. Moderate	Low vs. High	Moderate vs. High
HTA, n (%)	30 (96.8)	28 (93.3)	21 (70.0)	0.005	0.612	**0.005**	**0.020**
DM, n (%)	8 (25.8)	7 (23.3)	8 (26.7)	0.953	0.823	0.939	0.766
Heart disease, n (%)	5 (16.1)	6 (20.0)	6 (20.0)	0.904	0.694	0.694	1.000
HLP, n (%)	16 (51.6)	11 (36.7)	12 (40.0)	0.463	0.240	0.363	0.791
Ischemic stroke type, n (%)							
Arteriosclerotic	17 (54.8)	14 (46.7)	12 (40.0)	0.508	0.523	0.246	0.602
Cardioembolic	4 (12.9)	4 (13.3)	9 (30.0)	0.151	1.000	0.103	0.117
Lacunar	3 (9.7)	5 (16.7)	2 (6.7)	0.499	0.473	1.000	0.424
Other	7 (22.6)	7 (23.3)	7 (23.3)	0.997	0.994	0.944	1.000
NIHSS, med (min–max)	6 (2–16)	7 (3–14)	6 (2–21)	0.424	0.182	0.439	0.665

* for the level of significance of 0.05 according to chi-square test, Fisher’s exact test or Kruskal–Wallis test; Mann–Whitney U test was used for between-group comparisons where appropriate. Abbreviations: PA, physical activity, statistically significant *p*-values are shown in bold.

**Table 8:** Laboratory parameters immediately after first-ever ischemic stroke according to the level of physical activity.

Parameter	Low PA	Moderate PA	High PA	*p* *	Low vs. Moderate	Low vs. High	Moderate vs. High
Glycemia, Mean ± sd	7.2 ± 2.3	7.1 ± 2.3	6.5 ± 1.3	0.513	0.999	0.558	0.567
Total cholesterol, Mean ± sd	5.1 ± 1.9	4.7 ± 1.1	4.4 ± 1.7	0.654	0.627	0.888	0.919
HDL, med (min–max)	1.9 (0.9–2.4)	1.1 (1.0–1.3)	1.1 (0.9–1.3)	0.320	0.297	0.180	0.658
LDL, med (min–max)	3.6 (1.0–9.0)	3.9 (1.7–5.9)	3.5 (2.6–4.5)	0.970	0.897	0.796	0.827
Triglycerides	1.7 (0.9–5.2)	1.4 (0.7–5.2)	1.3 (0.7–3.8)	0.056	0.066	**0.027**	0.545
CRP, n (%)							
≤5	20 (80.0)	17 (68.0)	11 (61.1)	0.382	0.333	0.173	0.640
>5	5 (20.0)	8 (32.0)	7 (38.9)				
STA, mean ± sd	154.0 ± 29.1	152.4 ± 19.4	155.8 ± 22.8	0.898	0.970	0.966	0.889
DTA, mean ± sd	93.4 ± 17.1	90.6 ± 13.4	92.1 ± 13.9	0.809	0.793	0.961	0.943

* for the level of significance of 0.05 according to the one-way ANOVA and Tukey’s post hoc test. The Kruskal–Wallis test and Mann–Whitney U test were used for between-group comparisons; alternatively, the chi-square test was used where appropriate. Abbreviations: PA, physical activity; HDL, high-density lipoproteins; LDL, low-density lipoproteins, statistically significant *p*-values are shown in bold.

**Table 9:** Ischemic stroke consequences according to the level of physical activity.

Consequence	Low PA	Moderate PA	High PA	*p* *	Low vs. Moderate	Low vs. High	Moderate vs. High
Motor deficit side, n (%)							
Left	14 (45.2)	17 (56.7)	19 (63.3)	0.352	0.369	0.154	0.598
Right	17 (54.8)	13 (43.3)	11 (36.7)				
Functional deficit (hemiparesis/hemiplegia), n (%)	30 (96.8)	29 (96.7)	29 (96.7)	1.000	1.000	1.000	1.000
Functional deficit (aphasia/dysphasia), n (%)	6 (19.4)	10 (33.3)	10 (33.3)	0.376	0.215	0.215	1.000
Complications during hospitalization, n (%)	3 (9.7)	7 (23.3)	10 (33.3)	0.081	0.150	**0.024**	0.390
Early rehabilitation in hospital, n (%)	29 (93.5)	29 (96.7)	30 (100.0)	0.770F	1.000F	0.492F	1.000F
Time to rehabilitation, n (%)							
≤14 days	13 (41.9)	15 (50.0)	10 (33.3)	0.770	0.804	0.750	0.422
15–30 days	9 (29.0)	8 (26.7)	11 (36.7)				
>30 days	9 (29.0)	7 (23.3)	9 (30.0)				
Rehabilitation clinic stay, med (min–max)	34 (12–107)	45.5 (9–101)	40 (13–106)	0.585	0.348	0.988	0.395

* for the level of significance of 0.05 according to the Kruskal–Wallis test and Mann–Whitney U test; for between-group comparisons, the chi-square test was used where appropriate. Abbreviations: PA, physical activity, statistically significant *p*-values are shown in bold.

## Data Availability

The de-identified data supporting the findings of this study are available from the corresponding author upon request, subject to applicable ethical and institutional requirements. The data are not publicly available because the original dataset contains potentially identifiable participant information.

## Supplementary Materials

The following supporting information can be downloaded at: https://www.mdpi.com/article/10.3390/neurolint18100190/s1, Table S1. Disability and physical activity level.

## Abbreviations

The following abbreviations are used in this manuscript:

BI	Barthel Index
BPAQ	Baecke Physical Activity Questionnaire
CRP	C-Reactive Protein
DBP	Diastolic Blood Pressure
DM	Diabetes Mellitus
HDL	High-Density Lipoprotein
HLP	Hyperlipoproteinemia
HTN	Hypertension
IS	Ischemic Stroke
LDL	Low-Density Lipoprotein
LMM	Linear Mixed-Effects Models
MRI	Magnetic Resonance Imaging
NIHSS	National Institutes of Health Stroke Scale
OR	Odds Ratio
PA	Physical Activity
RR	Relative Risk
SBP	Systolic Blood Pressure
SPSS	Statistical Package for the Social Sciences
VIF	Variance Inflation Factor
