# Supplementary material for: Association Between Pre-Stroke Physical Activity Patterns Assessed by the Baecke Physical Activity Questionnaire and Functional Recovery After First Ischemic Stroke: A Barthel Index-Based Cohort Study
Source: Neurol Int. 2026 Oct 10;18(10):190. doi: 10.3390/neurolint18100190 (PMC13708209; doi:10.3390/neurolint18100190)
Supplement: Supplementary file 1 [file neurolint-18-00190-s001.zip › neurolint-4541413-supplementary.pdf]

**Supplementary Materials:**

Table S1 is showing disfunction level on admission, at discharge, and 3 months after discharge depending on the level of physical activity of first-ever ischemic stroke patients. The most patients on admission were moderately dependent (41%) with significant difference in distribution depending on the level of physical activity ($p = 0.021$). They were more common in patients with moderate than in low physical activity ($p = 0.007$). Patients who were slightly dependence on admission included 16% with significant difference depending on the level of physical activity ($p = 0.024$), and were more frequent in patients with low than with moderate physical activity ($p = 0.012$). On discharge, patients with slight dependence were common (47%), while there was a difference in distribution of patients with severe dependence according to the level of physical activity ($p = 0.017$), with greater frequency in patients with high than those with low and moderate physical activity ($p = 0.012$ and $p = 0.038$, respectively). Three months after discharge the largest portion of patients were slightly dependent (60%). There was no difference in the distribution of disfunction levels depending on the level of physical activity. Also, we evaluated the change of the number of disfunction levels in time from admission, to discharge, up to 3 months after discharge. The frequency of totally dependent patients did not change through the time. The number of severe dependent patients decreased between admission and discharge ($p < 0.001$) and between admission and 3 months after discharge ($p < 0.001$). Moderate dependency decreased from discharge to 3 months after it ($p = 0.049$). Number of slightly dependent patients increased between admission and discharge ($p < 0.001$), discharge and 3 months after it ($p = 0.002$), as well as from admission up to 3 months after discharge ($p < 0.001$).

Table S1. Disability and physical activity level

| Disfunction level according to BI, n (%) | Physical activity | | | *p* | Low vs. Moderate | Low vs. High | Moderate vs. High |
|---|---|---|---|---|---|---|---|
| | Low | Moderate | High | | | | |
| On admission | | | | | | | |
| Total dependency (0-20) | 1(3.2) | 2 (6.7) | 3 (10.0) | 0.522F | 0.612F | 0.354F | 1.000F |
| Severe dependency (21-60) | 13 (41.9) | 9 (30.0) | 11 (36.7) | 0.624 | 0.332 | 0.674 | 0.584 |
| Moderate dependency (61-90) | 8 (25.8) | 18 (60.0) | 11 (36.7) | **0.021** | **0.007** | 0.360 | 0.071 |
| Slight dependency (91-99) | 9 (29.0) | 1 (3.3) | 5 (16.7) | **0.024F** | **0.012**F | 0.251 | 0.195F |
| At discharge | | | | | | | |
| Total dependency (0-20) | 1 (3.2) | 1 (3.2) | 0 (0.0) | 1.000F | 1.000F | 1.000F | 1.000F |
| Severe dependency (21-60) | 1 (3.2) | 2 (6.7) | 8 (26.7) | **0.017F** | 0.612F | **0.012F** | **0.038** |
| Moderate dependency (61-90) | 14 (45.2) | 12 (40.0) | 9 (30.0) | 0.466 | 0.684 | 0.222 | 0.417 |
| Slight dependency (91-99) | 15 (48.4) | 15 (50.0) | 13 (43.3) | 0.864 | 0.900 | 0.692 | 0.605 |
| 3 months after discharge | | | | | | | |
| Total dependency (0-20) | 1 (3.2) | 0 (0.0) | 0 (0.0) | 1.000F | 1.000F | 1.000F | NA |
| Severe dependency (21-60) | 1 (3.2) | 2 (6.7) | 6 (20.0) | 0.091F | 0.612F | 0.053F | 0.254F |
| Moderate dependency (61-90) | 9 (29.0) | 9 (30.0) | 8 (26.7) | 0.958 | 0.934 | 0.837 | 0.774 |
| Slight dependency (91-99) | 20 (64.5) | 19 (63.3) | 16 (53.3) | 0.621 | 0.923 | 0.375 | 0.432 |
